# Thermal damage of tungsten-armored plasma-facing components under high heat flux loads

**DOI:** 10.1038/s41598-020-57852-8

**Published:** 2020-01-28

**Authors:** Shuming Wang, Jiangshan Li, Ye Wang, Xiaofang Zhang, Ruiping Wang, Yanru Wang, Jian Cao

**Affiliations:** 1 Department of Materials Science and Engineering, University of Science &Technology Beijing, Beijing, 100083 China; 2 State Key Laboratory of Advanced Welding and Joining, Harbin Institute of Technology, Harbin, 150001 China

**Keywords:** Metals and alloys, Power stations

## Abstract

Fusion energy is expected as a promising candidate for alternative next generation energy. For fusion reactor, the plasma facing components (PFCs) are the most critical components to achieve this goal. PFCs will suffer severe thermal shock due to repective cyclic high heat flux (HHF) loads. This paper investigates the effects of thermal shock and damage behavior of tungsten armored PFCs under steady, transient and combined thermal loads. The distribution of stress field is analyzed, and crack initiation is predicted using the extended finite element method (XFEM). The unique features of thermal-mechanical behavior of tungsten armored PFCs under simulated service condition are discussed. The dominant factor of the cracking of the tungsten armor is the brittleness of tungsten below ductile-to-brittle transition temperature (DBTT). Under the steady loads, the cracking position is apt to near the interface of tungsten armor and the interlayer, and the threshold of cracking is between 14 MW/m^2^ and 16 MW/m^2^. With 6 MW/m^2^ steady loads, applying 1 ms duration of transient load, the cracking threshold is between 0.2 GW/m^2^ to 0.4 GW/m^2^. The depth of cracking increases from 100 um to 500 um with the transient load increasing from 0.4 GW/m^2^ to 1.0 GW/m^2^. Researches are useful for the design and structural optimization of tungsten-armored PFCs, and the long-term stable operation of further reactor.

## Introduction

In thermonuclear fusion reactor, PFCs are crucial to withstand high temperature plasma irradiation and timely transfer heat away to protect vacuum chamber walls and internal components. The PFCs are generally composed of plasma facing materials (PFMs), interlayer and heat sink materials^1,2^. With the development of fusion reactors, candidate PFMs have undergone a transition from low-Z carbon-based or beryllium materials to high-Z tungsten^3,4^. As tungsten has many unique properties such as low sputtering erosion and tritium retention, high melting point and moderate thermal expansion^5^, it has been choosing as the main divertor PFMs in ITER and has been foreseen as the most suitable candidate for the first wall in demonstration fusion reactor (DEMO) or future fusion reactors^6–8^.

In addition, tungsten monoblocks in the divertor vertical target are designed as the high heat flux handling unit where heat loads are maximal^9^. Therefore, the study on the thermal shock performance and damage behavior of tungsten divertor monoblock is of great significance to the safe operation of the reactor. This paper aims to systematically investigate the damage behavior of ITER tungsten divertor monoblock under steady state and ELM-like thermal shock loads, mainly including the plastic deformation and cracking behavior of tungsten armor.

## FE Model

### Geometry, FE mesh and materials

**Figure 1:**
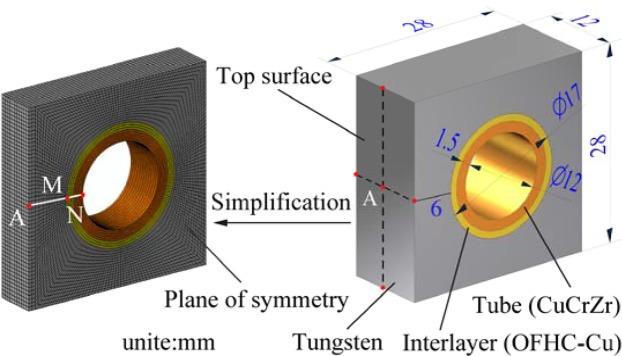
Sketch map of the model size and the FE mesh of the model.

We design the monoblock and build the model as shown in Fig. 1^8,10–12^. Both width and height of the selected monoblock are 28 mm, the axial length is 12 mm and the armor thickness is 6 mm. The CuCrZr alloy has been chosen as the heat sink material because of its good irradiation resistance and high thermal conductivity, and its tube diameters are 12/15 mm (ID/OD). The oxygen free high conductivity copper (OFHC-Cu) with a thickness of 1 mm is selected as an interlayer to reduce the thermal stress caused by the thermal expansion mismatch of PFMs and heat sink material CuCrZr^13,14^. The thermal physical properties of the selected materials are listed in Table 1 which is created based on ITER material properties handbook^15^ and related documents^16–18^.

**Table 1:** Thermal physical properties of the selected materials at considered temperature.

Material	Temperature (°C)	Thermal conductivity (W·m^−1^·K^−1^)	Coefficient of thermal expansion (10^−6^ K^−1^)	Young’s modulus (GPa)	Poisson’s ratio	Yield strength (MPa)	Tangent modulus (GPa)
W	20	173	4.5	398	0.28	1360	1.3
	200	156	—	396	—	1154	—
	500	133	4.7	390	—	854	1
	800	118	—	379	0.29	604	—
	1000	111	5.1	368	—	465	0.8
	1500	101	5.6	333	0.30	204	—
	1800	99	5.8	306	—	103	—
OFHC-Cu	20	403	16.7	125	0.34	69	1.5
	200	392	17.2	115		60	1.3
	400	379	17.8	100		48	0.9
	700	360	18.9	70		30	0.6
CuCrZr	20	326	16.7	128	0.32	293	0.9
	250	343	17.2	118	0.42	257	0.7
	500	348	18.2	103	0.52	195	0.6

The FE package ABAQUS^19^ was employed for the simulations using quadratic brick elements of 20 nodes. According to the symmetry of the sample, the 1/2 simplified FE model was used, as shown in Fig. 1, with a total of 61,152 elements.

### Loads and boundary conditions

The ITER-relevant cooling condition is selected: coolant water for 10 m/s, 4.0 MPa at 100 °C and perfect thermal contacts at the interfaces were assumed^20^. In the analysis of FE, different thermal loads were uniformly applied to the top surface of the monoblock and 100 °C was set as the initial temperature. Stefan-Boltzmann’s law was adopted to calculate the radiation loss, and emissivity of loaded surface was defined as 0.3^11^. The coefficient of convection heat transfer (CCHF) between the cooling tube and the coolant water was 110 kW/(m^2^·K), which was calculated using the well-known Dittus-Boelter formula^21^. Finally, symmetric boundary conditions were applied based on simplification, and a pressure of 4.0 MPa was applied to the inner surface of cooling tube to simulate the pressure caused by cooling water. The boundary conditions were as follows: mechanical constraint in all directions at bottom face of W monoblock, the side surface of interlayer and tungsten armor is free; the thermal conduction between a heated tungsten armor and the neighbor armor is negligible; coolant temperature at 100 °C. It should be noted that all components are bonded to each other perfectly for simplifying the FE analysis.

## Steady Heat Flux Simulation

During the operation of ITER, high-temperature plasma will deposit energy on the surface of divertor target through thermal radiation and particle collision. During some transient event (up to 10 s), steady load can reach to 10~20 MW/m^2^ at tungsten monoblock in the vertical target^9^. To study the real working conditions of the monoblock in the fusion device, heat flux loads which range from 6 MW/m^2^ to 20 MW/m^2^ were applied on the top surface of tungsten monoblock.

### Thermal and mechanical simulations

**Figure 2:**
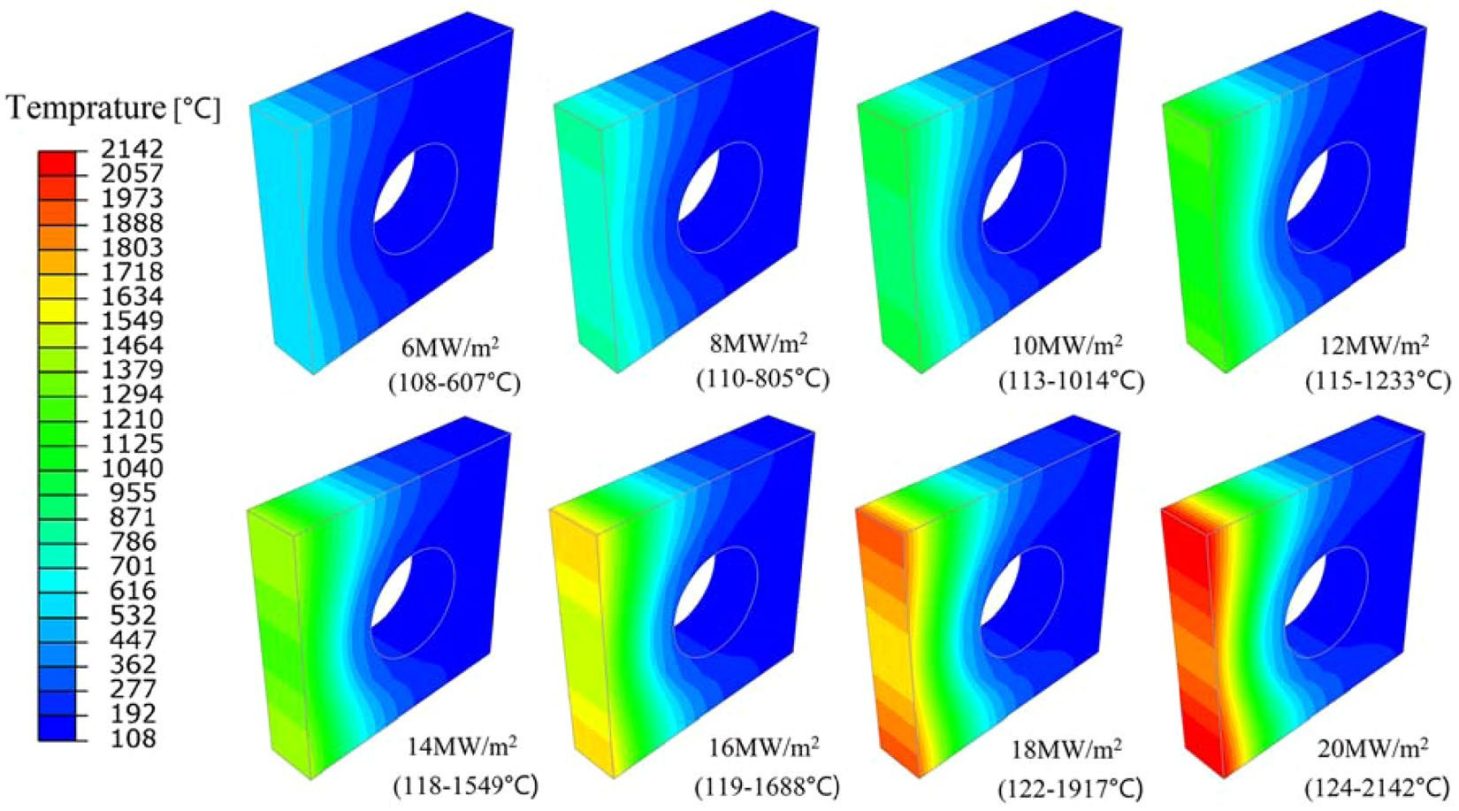
Temperature contour plots under different steady state loads.

**Figure 3:**
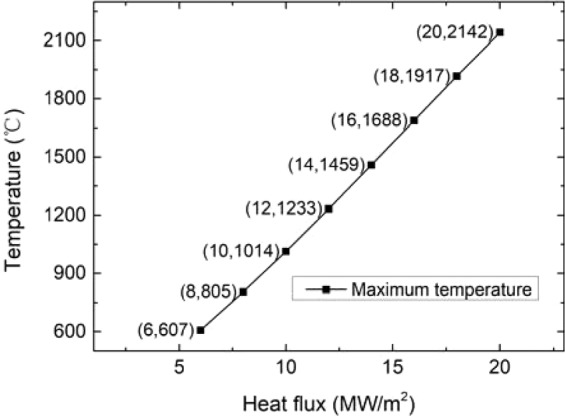
The highest temperature values at different steady state loads.

The temperature distribution of the monoblock under different steady loads is illustrated in Fig. 2. With different heat flux loads, the temperature at the bottom of the sample is relatively low, less than 200 °C, and the highest temperature appears at the top surface of the monoblock. Figure 3 shows the highest temperature at different heat flux, and a nearly linear relationship between different heat flux densities and the highest temperatures they produced is demonstrated.

**Figure 4:**
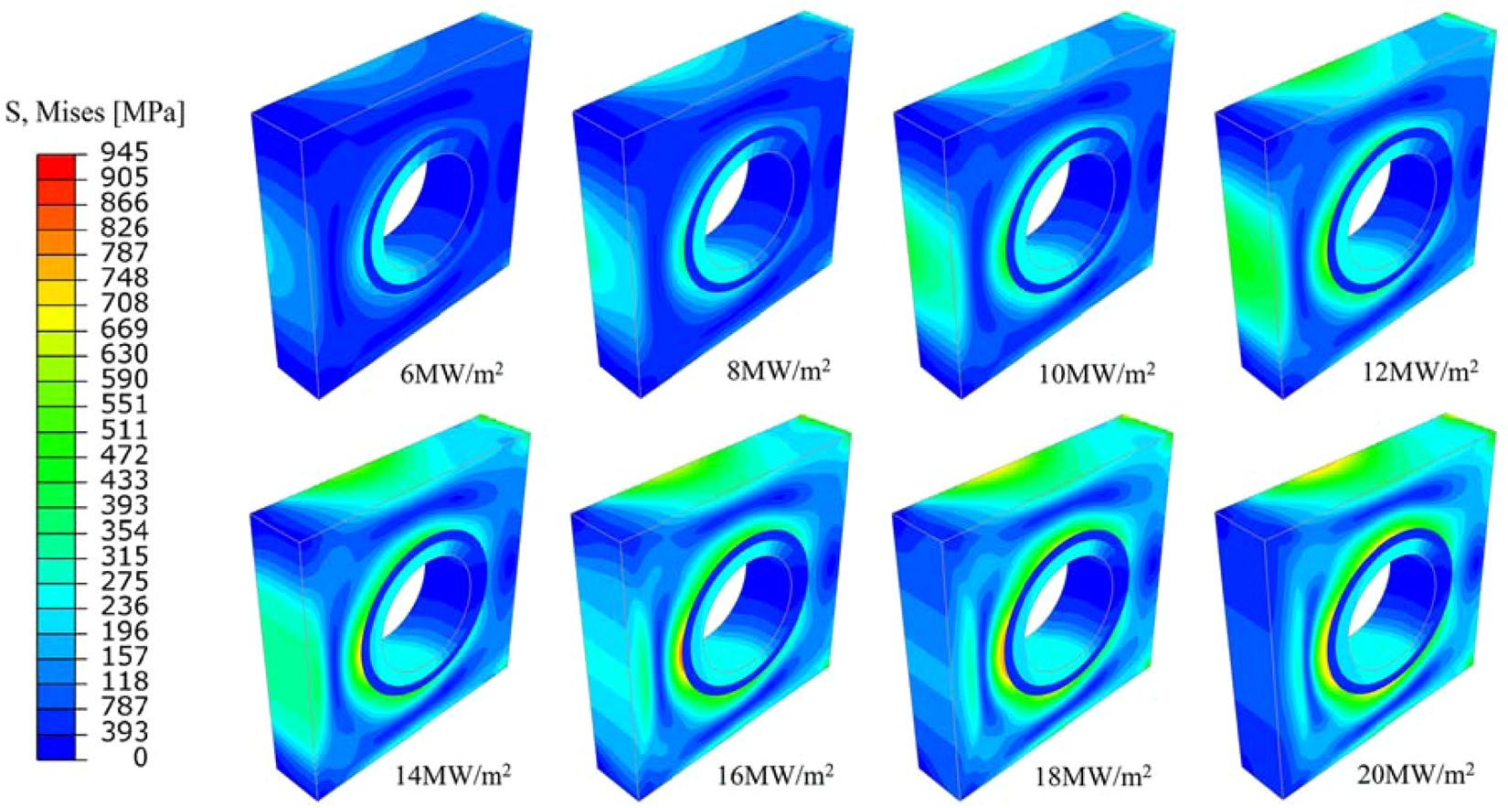
Von Mises equivalent stress contour plots under different steady state loads.

The stress distribution of monoblock at different steady state loads is shown in Fig. 4. Because of the gradual accumulation of heat, as the heat flux density increases from 6 MW/m^2^ to 12 MW/m^2^, thermal stress of the top surface of tungsten armor increases gradually. When the heat flux is higher than 12 MW/m^2^, the thermal stress exceeds the yield strength, causing plastic deformation and the value of stress gradually decreases. The position with highest stress at different steady loads is always near the junction between tungsten armor and interlayer.

The Mises yield criterion can be used to determine whether or not the material yields which is resulted from plastic deformation^22^.

1 $${{\rm{\sigma }}}_{{\rm{e}}}=\sqrt{\frac{\,{({{\rm{\sigma }}}_{1}-{{\rm{\sigma }}}_{2})}^{2}+{({{\rm{\sigma }}}_{2}-{{\rm{\sigma }}}_{3})}^{2}+{({{\rm{\sigma }}}_{3}-{{\rm{\sigma }}}_{1})}^{2}}{\,2}}\ge {{\rm{\sigma }}}_{s}$$

**Figure 5:**
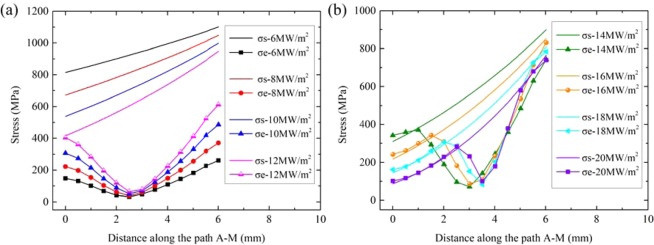
σe and σs on the path A-M under different steady-state heat flux loads: (**a**) 6–12 MW/m^2^; (**b**) 14–20 MW/m^2^.

**Figure 6:**
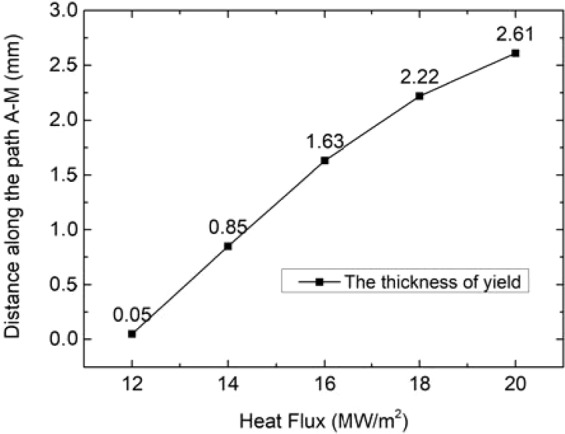
Plastic deformation thickness of tungsten armor under different steady state loads.

Where σ_e_ is Mises equivalent stress, σ_1_ is the first principal stress, σ_2_ is the second principal stress, σ_3_ is the third principal stress, σ_s_ is the Yield Strength. Under different heat flux loads, the σ_s_ corresponding to the temperature distribution on the path A-M (Fig. 1) and the σ_e_ on the path A-M are represented in one figure, as shown in Fig. 5. 12 MW/m^2^ is the threshold of plastic deformation. In the range of 6–10 MW/m^2^, σs is always greater than σe, which means the material is not plastically deformed. When the steady state load is greater than 12 MW/m^2^, plastic deformation occurs on the surface of the armor. Figure 6 shows the thickness of plastic deformation under loads between 12 MW/m^2^ and 20 MW/m^2^.

### XFEM simulations

XFEM, an extension of the conventional finite element method, is used to simulate crack initiation and propagation^23^. The criterion of maximum principal stress (MPS) and energy-based damage evolution law are used. Once the principal stress exceeds the maximum allowable value, cracks will be initiated. Considering that the formation of cracks is mainly due to the brittleness of tungsten below DBTT^24^, MPS is defined as 700–900 MPa according to the corresponding temperature (400–700 °C), which is estimated from the value of maximum tensile strength of tungsten in the temperature near DBTT. In the process of crack propagation, a certain amount of energy is released. According to the basic hypothesis of energy release rate criterion, when the maximum energy release rate reaches a critical value, the cracks become unstable and begin to expand. The critical energy release rate is defined as 0.25 mJ/mm^2^, referenced the researches performed by Gludovatz *et al*.^25^.

**Figure 7:**
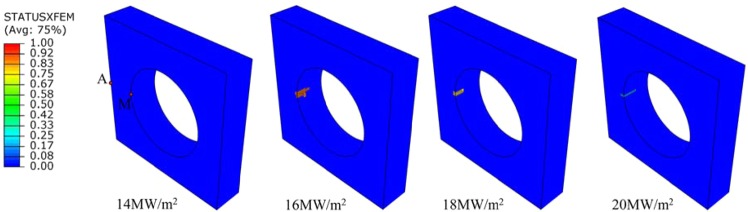
XFEM simulations for different steady-state loads. The value of STATUSXFEM is 1.0 characterizes an opened crack. The values smaller than 1.0 present that cracks require additional energy to be opened. The heat sink layer and the interlayer are not shown in the plots.

**Figure 8:**
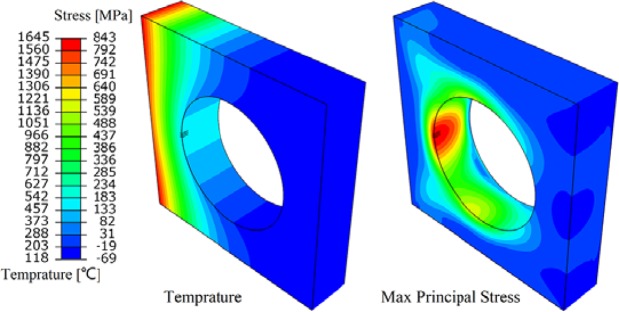
Crack initiation time of 16 MW/m^2^. Temperature contour plots (left) and maximum principal stress contour plots (right).

Cracking under different steady loads is shown in Fig. 7, when steady load value is greater than or equal to14 MW/m^2^, cracking is predicted. Therefore, the threshold of cracking under steady state load is between 14 MW/m^2^ and 16 MW/m^2^. The thermal expansion mismatch of tungsten armor and interlayer causes the stress concentration at the junction of those two layers. If the stress exceeds tensile strength of tungsten it may be cracked. Taking the 16 MW/m^2^ crack initiation time as an example (Fig. 8), the maximum value of the principal stress exceeds the corresponding MPS (700 MPa), crack initiations are predicted.

## Transient Heat Flux Simulation

During the operation of fusion reactor, The PFCs targets suffers not only steady state thermal loads but strong transient loads such as edge localized modes (ELMs, about 1 MJ m^−2^) and disruptions (several 10 MJ m^−2^)^26^. In this study, a transient heat flux was applied to the top surface of tungsten while sample was under different steady loads conditions to simulate the damage on the monoblock caused by ELM-like transient thermal shock loads. Then all thermal loads are removed and the monoblock is cooling down. The boundary conditions were the same as in the steady simulation.

### Thermal and mechanical simulations

**Figure 9:**
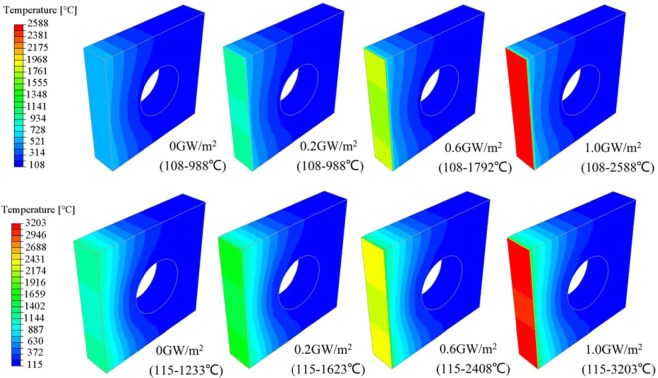
Temperature contour plots after 1.0 ms of different transient thermal shocks is applied on the basis of steady-state load. Steady state load is 6 MW/m^2^ (above). Steady state load is 12 MW/m^2^ (below).

**Figure 10:**
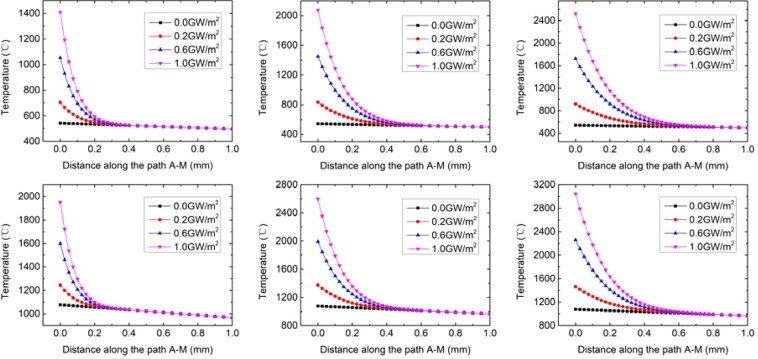
Temperature distribution on the path A-M after the application of transient loads for 0.2 ms, 0.6 ms and 1.0 ms. Steady state load is 6 MW/m^2^ (above). Steady state load is 12 MW/m^2^ (below).

Figure 9 shows the temperature distribution with different transient loads of 1.0 ms under steady loads of 6 MW/m^2^ and 12 MW/m^2^ (0 GW/m^2^ means no transient load). Applying different transient loads, temperature changing on the path A-M demonstrates the effects of transient load on the tungsten armor. The impacting depth of different transient loads with loading times of 0.2~1.0 ms on tungsten armor is 0.4~0.8 mm (Fig. 10). A remarkable fact is that when the pulse duration is constant, the impacting depth of different transient loads on tungsten armor is almost the same, in other words, the impacting depth on tungsten armor only increases as the pulse duration increases. In addition, the greater the load value is, the higher the temperature gradient on the path is.

It should be noted that if the transient load and the pulse duration are fixed, the increase of the surface temperature of tungsten armor would be a fixed value which is unrelated to the steady state load value (base temperature). When transient load is 1 GW/m^2^ for 1 ms, the temperature increasing of the top surface of tungsten armor is about 1950 °C. Therefore, to avoid the surface temperature of tungsten armor reaching melting point (3420 °C), the surface temperature of tungsten armor under steady load should be lower than 1470 °C. In other words, steady load should lower than 14 MW/m^2^.

**Figure 11:**
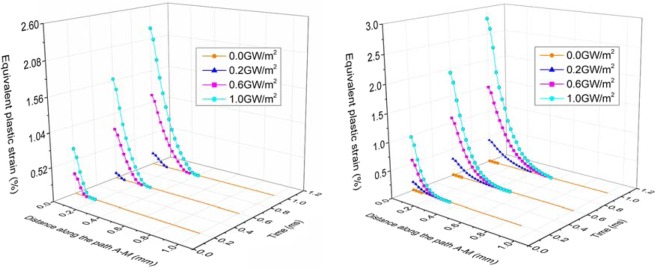
Equivalent plastic strain on the path A-M after the application of transient loads for 0.2 ms, 0.6 ms and 1.0 ms. Steady-state load is 6 MW/m^2^ (left). Steady-state load is 12 MW/m^2^ (right).

The effect of different transient loads combined with steady loads of 6 and 12 MW/m^2^ on the plastic deformation of the tungsten armor is represented in Fig. 11. Under the base condition of steady load of 6 MW/m^2^, when the pulse duration of transient load is 0.2 ms and the transient load value is 0.6 GW/m^2^ or 1.0 GW/m^2^, the surface of the tungsten armor is plastically deformed. When the pulse duration of transient load is 0.6 ms or 1.0 ms and the transient load value is 0.2 GW/m^2^, 0.6 GW/m^2^ or 1.0 GW/m^2^, the surface of the tungsten armor is plastically deformed. Under the steady load of 12 MW/m^2^, the top surface of tungsten armor has undergone plastic deformation. As the transient load and the pulse duration increase, the thickness and the degree of plastic deformation increase.

### XFEM simulations

Table 2 shows the situation of cracking within different transient loads combined steady loads of 6 and 12 MW/m^2^. “Y” represents that the cracking is predicted and “N” is not. The dash line in the table represents the threshold of the load value and the pulse duration of cracking.

**Table 2:** The situation of crack initiation with different transient loads applied under steady-state loads.

	6 MW/m^2^			12 MW/m^2^		
	+0.2 GW/m^2^	+0.6 GW/m^2^	+1.0 GW/m^2^	+0.2 GW/m^2^	+0.6 GW/m^2^	+1.0 GW/m^2^
0.0 ms	N	N	N	N	N	N
0.2 ms	N	N	N	N	Y	Y
0.6 ms	N	Y	Y	N	Y	Y
1.0 ms	N	Y	Y	N	Y	Y

**Figure 12:**
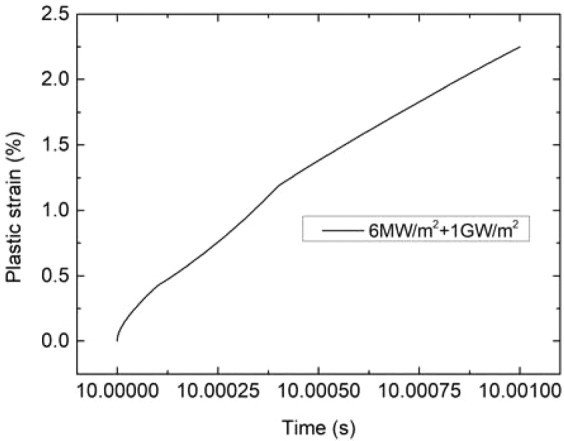
The increase of the A point plastic strain during the application of transient load.

**Figure 13:**
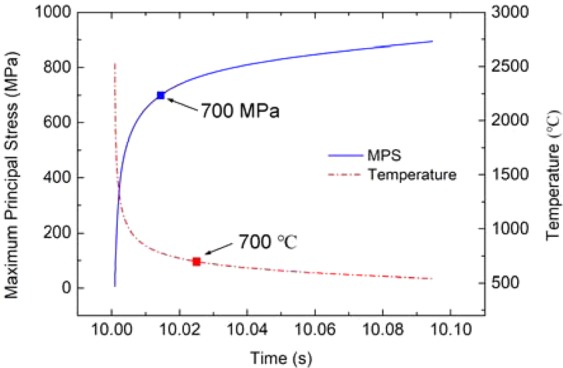
The change of temperature and the maximum principal stress of A point over cooling period.

In process of the transient load thermal shock, the tungsten surface temperature rises rapidly and irreversible expansion occurs. The plastic strain increasing of the “A” point (cracking area) during the application of transient load of 1 GW/m^2^ for 1 ms combined with steady load of 6 MW/m^2^ is shown in Fig. 12. And Fig. 13 shows the changes of temperature and the maximum principal stress of “A” point during cooling period. It can be found that the principal stress has already exceeded the critical MPS (700 MPa) before the temperature drop down to DBTT (~700 °C). This result validates the illustration of the experiment on cracking of tungsten under single pulse thermal shock loads^24^. During the cooling period, the plastic deformation remains in the load application area, which hinders the contraction of the elastic deformation and generates tensile stress. When the temperature drops below DBTT, tungsten is in brittle state. If the stress exceeds the tensile strength, cracking will occur.

**Figure 14:**
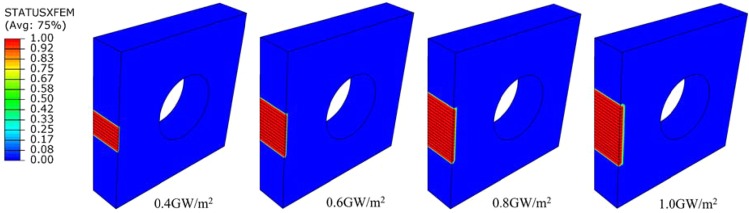
Cracking in different transient loads for 1 ms under steady state load of 6 MW/m^2^.

**Figure 15:**
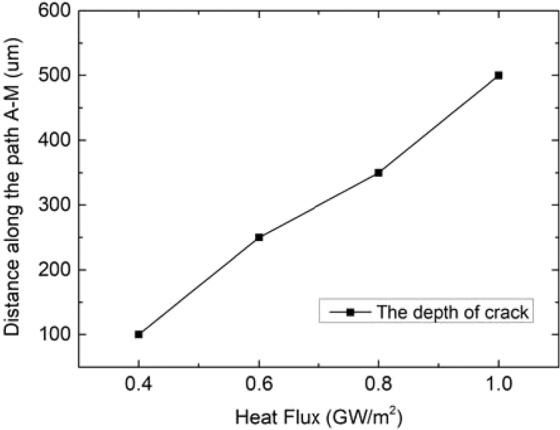
The depth of crack in different transient loads for 1 ms under steady state load of 6 MW/m^2^.

Applying different transient loads for 1 ms under steady load of 6 MW/m^2^, cracks around the middle area of top surface of monoblocks parallel to the coolant tube axis are show in Fig. 14. When the pulse duration of transient loads is fixed, the higher the transient load applied, the larger the plastic strain on the path A-M will be, and the deeper the cracking depth will be. The cracking depth increases from 100 um to 500 um when transient loads increases from 0.4 GW/m^2^ to 1.0 GW/m^2^, as shown in Fig. 15.

## Conclusions and Discussions

The damage behavior of ITER tungsten divertor monoblock under steady and ELMs-like transient thermal shock loads is investigated using the method of FEM and XFEM. The main results are summarized as follows:

Under steady loads, Plastic deformation of the top surface of tungsten armor is found at 12 MW/m^2^ and the threshold of tungsten armor crack initiation is between 14 MW/m^2^ and 16 MW/m^2^, the thermal expansion mismatch between tungsten armor and interlayer causes stress concentration which is the driving force for cracking. The cracking position is near the interface of tungsten armor and interlayer. In fact, there is a complex interface between tungsten and the interlayer, therefore, it should be noted that the results at the interface between tungsten and the interlayer are indicative and need to be verified through further experiments. This result reflects the problem that stress concentration caused by the mismatch of thermal expansion coefficient should be noted.

During the cooling stage, tensile stress caused by plastic deformation is the driving force for cracking. However, under steady loads, the plastic deformation of the surface is insufficient to cause cracking. Based on steady loads, applying a transient load, the amount of plastic deformation on the surface of tungsten armor will increase. When the plastic strain increases to a certain extent, cracking will occur on the top surface of the tungsten. And the larger the plastic strain is, and the greater the depth of cracking will be.

The crack caused by a single pulse is mainly due to the brittleness of tungsten below the DBTT. When the base temperature is set above a certain threshold (DBTT), both the simulation^5,27^ and experiment^24,28^ results implied that there is no crack on tungsten with single pulse loading. Under this circumstance, the fatigue failure caused by the cyclic load becomes the main factor of cracking and the numerical simulation of the fatigue failure behavior of tungsten monoblock under cyclic loading should be considered.
